# P75 nerve growth factor receptors modulate development of GnRH neurons and olfactory ensheating cells

**DOI:** 10.3389/fnins.2013.00262

**Published:** 2013-12-27

**Authors:** Franca Raucci, Jean D. Tiong, Susan Wray

**Affiliations:** Cellular and Developmental Neurobiology Section, National Institute of Neurological Disorders and Strokes, National Institutes of HealthBethesda, MD, USA

**Keywords:** LHRH neurons, olfactory ensheathing cells, migration, olfactory system, development

## Abstract

Temporal and spatial localization of nerve growth factor receptor (p75NGFR) in the developing olfactory system and gonadotropin-releasing hormone-1 (GnRH) system was characterized and its role analyzed using p75NGFR null mice and nasal explants. Prenatally, p75NGFR was expressed by GnRH neurons and olfactory ensheathing cells (OECs). In p75NGFR null mice, no change in the number of GnRH cells was detected as compared to wild-type. However, in null mice, a shift in the distribution of GnRH neurons was found, with a small population of GnRH cells migrating further caudally toward the median eminence. Additionally, a reduction of both GAD67 positive olfactory axons and GFAP positive OEC fibers occurred. Acute administration of a p75NGFR blocker to GnRH cells maintained *in vitro* increased migration rate, consistent with the change in distribution detected in p75NGFR null mice. Chronic inhibition of p75NGFR caused an attenuation of olfactory axon fasciculation and a decrease in OEC density, again mimicking the changes detected in null mice. However, a reduction in GnRH cell number was found after chronic treatment that not observed in KO animals suggesting indirect changes occur during chronic treatment *in vitro* and/or a compensatory mechanism occurs *in vivo* that prevents loss of GnRH neurons in the absence of p75NGFR.

## Introduction

During development, gonadotropin releasing hormone-1 (GnRH) neurons and olfactory ensheathing cells (OECs) originate from nasal placode and then migrate from the olfactory epithelium to the developing central nervous system in association with olfactory axons (Wray, 2010; Forni et al., 2011). In patients with Kallmann's Syndrome, anosmia, and absence of puberty are attributed to defects in GnRH neuronal migration and olfactory sensory axon targeting. In patients with idiopathic hypogonadotropic hypogonadism (IHH), reproductive dysfunction is not accompanied by anosmia. While many IHH defects are attributed to postnatal events, some appear to be developmental in origin (Wray, 2010). These data imply that cues may act directly on GnRH neurons or indirectly via disruption of olfactory components to cause migrational defects in the GnRH system.

p75 neurotropin receptors (p75NGFR), mediate multiple cellular functions including cell survival, apoptosis, proliferation, differentiation, regeneration, axonal extension, neurite outgrowth, or collapse (Koh and Higgins, 1991; Tanaka et al., 1997; Yamashita et al., 1999, 2005; Krygier and Djakiew, 2002; Hasegawa et al., 2004; Arevalo and Wu, 2006; Blochl and Blochl, 2007; Cragnolini and Friedman, 2008). OECs express p75NGFR (Vickland et al., 1991; Gong et al., 1994), but little is known about its function in these cells (Su et al., 2007; Cragnolini and Friedman, 2008). Homozygote p75NGFR null mice display several defects in the nervous system, such as impaired innervation by peripheral sensory neurons (Lee et al., 1992). Comparison of peripheral axons in p75NGFR^−/−^ and wild-type mice (excluding the olfactory system, Bentley and Lee, 2000) showed severe stunting and poor arborization, and decreased Schwann cell migration between E11.5 and E14.5. Within the olfactory system of p75NGFR^−/−^ mice, ectopic glomerular-like tufts were found postnatally, but no changes were reported prenatally after examination of Haematoxylin and Eosin stained sections (Tisay et al., 2000). Based on these results, Tisay et al. (2000) concluded that p75NGFR is not essential for the assembly of the olfactory nerve during embryogenesis but indirectly, via OECs, regulates axon growth postnatally.

This study examined the role of p75NGFR in development of the GnRH/olfactory system in mouse. During prenatal development, p75NGFR was detected in a subpopulation of migrating GnRH neurons and the majority of OECs. GnRH cell number was similar in both WT and p75NGFR KO mice. However, a shift in the distribution of GnRH neurons occurred in the p75NGFR KO mice, with a small population of GnRH cells migrating further in the hypothalamus, closer to the median eminence. In addition, there was a reduction in GAD67 positive olfactory axons as well as GFAP positive OECs in nasal regions. p75NGFR functional blocking antibody acutely applied to GnRH cells maintained in nasal explants increased migration of GnRH cells, consistent with the increase in GnRH cells that migrated caudally in the brain of p75NGFR^−/−^ mice. Chronic inhibition of p75NGFR reduced GnRH cell number, decreased fasciculation of olfactory axons and produced a change in OEC morphology. Attenuated olfactory axon fasciculation was consistent with findings in the KO mouse. However, the loss of GnRH cells *in vitro* after chronic blockage of p75NGFR is in contrast to that observed in the KO animal and suggests that indirect changes occur during chronic treatment *in vitro* and/or a compensatory mechanism occurs *in vivo* that prevents the loss of GnRH neurons.

## Materials and methods

### Animals

All mice were killed in accordance with National Institutes of Health, National Institute of Neurological Stroke and Disorders guidelines. NIH Swiss embryos were collected at E11.5, E12.5, E14.5, and E17.5 (plug day, E0.5) and immediately frozen and stored (−80°C). p75NGFR-deficient mice (^−/−^) were kindly provided by Dr. B. Lu (Laboratory of Cellular and Synaptic Neurophysiology, NICHD). Embryos (E16.5) and adult brains of p75NGFR^−/−^ and wild-type (WT) mice were harvested, frozen and stored (−80°C) until processed for immunocytochemistry.

### Nasal explants

Nasal regions were cultured as described previously (Fueshko and Wray, 1994). Briefly, nasal pits of E11.5 NIH Swiss mice were isolated under aseptic conditions and adhered onto coverslips by a plasma (Cocalico Biologicals, Reamstown, PA)/thrombin (Sigma, St. Louis, MO) clot. Explants were maintained in defined serum-free medium (SFM) at 37°C with 5% CO_2_. On culture day 3, fresh media containing fluorodeoxyuridine (8 × 10^−5^ M; Sigma) was given to inhibit proliferation of dividing olfactory neurons and non-neuronal explant tissue. On culture day 6, and every 2 days thereafter, the explants received fresh SFM.

### Transcript analyses on single GnRH cell

cDNAs from whole explants and single GnRH cells previously isolated from explants were used in this study (Kramer et al., 2000a; Giacobini et al., 2004; Temple and Wray, 2005; Toba et al., 2008). 3′ UTR based primers (Table 1) were used in PCR to determine expression of p75NGFR, TrKA, TrKB, and TrKC in single GnRH cells at three time points [3, 7, and 14 days *in vitro* (*div*)]. Primers for NGF, BDNF, NT3, and NT4,5 (Table 1) were used on cDNA from whole explants. All primers were designed from GenBank sequences and screened using BLAST (http://blast.ncbi.nlm.nih.gov/Blast.cgi). cDNAs prepared from adult mouse brain and E17.5 embryo head were used as controls. Amplified PCR products were run on a 1.5% agarose gel.

**Table 1:** **Summary of primers sequences**.

**Primers**	**Sequences**
p75 NGFR	F1 5′-TGCAATTAGTAGAAGGACCCCACC-3′
	R1 5′-TACACAGGATAGCAAAGGGGA-3′
TrkA	F1 5′-TATGGAAAGCAGCCCTGGTACC-3′
	R1 5′-ACTTGAATGTGGTAGCTCCTGG-3′
TrkB	F1 5′-TTCAGGAAAACCCGAGTC-3′
	R1 5′-TTCAGGAAAACCCGAGTCC-3′
TrkC	F1 5′-GGAGCCATCTACTAGTGAAG-3′
	R1 5′-CAAGCATTTATACTCTGTTGC-3′
NGF	F1 5′-TTTCAACAGGACTCACCGGAGC-3′
	R1 5′-CTGTC=GTCTATCCGGATGAACC-3′
BDNF	F1 5′-CCATGGGTTACACCAAGGAAG-3′
	R1 5′-GTCTGCATTACATTCCTCG-3′
NT3	F1 5′-GCAGAACATAAGAGTCACCGAGG-3′
	R1 5′-TCATCAATCCCCCTGCAACC-3′
NT4/5	F1 5′-TGCGTCAGTACTTCTTCGAGACG-3′
	R1 5′-TGTGTCGATCCGAATCCCAGC-3′

### Immunocytochemistry (ICC)

Primary antisera used were against GnRH [polyclonal SW-1, 1:3000, (Wray et al., 1988)], peripherin (1:2000, Chemicon, Temecula, CA), p75NGFR (1:5000, Chemicon), nestin (1:3000, Kramer et al., 2000b), S100 (1:3000–4000, Dako, Glostrup, DN), GAD67 (1:8000, gift from L. J. Kopin; Oertel et al., 1981; Lee et al., 2008), PSA-NCAM (1:4000, Millipore, Billerica, MA), GFAP (Glial Fibrillary Acidic Protein, 1:18, Incstar, Stillwater, MN), Sox-10 (1:400, Forni et al., 2011), neurophysin (RN2, 1:12000, Wray et al., 1988), S100 (1:1000, Dako, Glostrup, DN), and Caspase-3 (1:200, Millipore, Billerica, MA). Alexa Fluor-546 phalloidin, (1:40, Molecular Probe, Eugene, OP) was used to visualize actin filaments.

**Figure 1:**
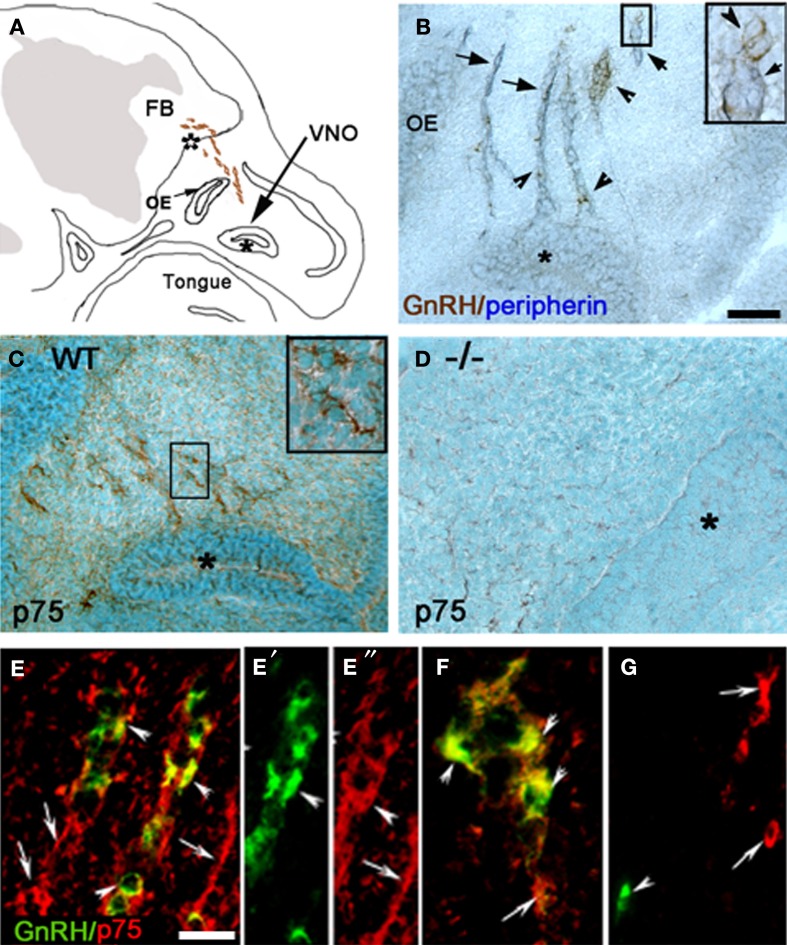
**GnRH neurons express p75NGFR. (A)** Camera lucida drawing of an E12.5–E14.5 mouse head showing the localization of GnRH neurons (brown dots) in the nasal region. **(B)** Sagittal section from an E12.5 mouse immunostained for GnRH (brown, arrowhead) and peripherin (blue, arrow; insert = higher magnification of boxed region). Both cells and fibers are detected leaving the vomeronasal organ (VNO, marked by asterisk). **(C)** Immunostaining for p75NGFR (p75, brown) spatially overlapped with GnRH neurons and olfactory fiber tracks/pathways. **(D)** Sagittal section from an E16.5 p75NGFR mutant embryo (^−/−^) stained with the p75NGFR antibody. No p75NGFR staining was observed. **(E–E″)** Double-labeling for GnRH (green) and p75NGFR (red) showed that the majority of migrating GnRH neurons emerging from the VNO co-expressed the receptor (arrowheads), but p75NGFR immunoreactivity was also localized to structures other than GnRH cells (arrows). **(F)** Double-labeling for GnRH (green) and p75NGFR (red) showed that the majority of GnRH cells were still p75NGFR positive (arrowheads) at the nasal-forebrain junction; some p75NGFR immunopositive/GnRH negative elements were also present (arrows). **(G)** Adult mouse brain (hypothalamic area) stained for GnRH (green) and p75NGFR (red). GnRH positive cells (arrowhead) did not colocalize with p75NGFR, although p75NGFR positive cells were present in nearby areas (arrows). OE = Olfactory epithelium, FB = forebrain; white asterisk = nasal forebrain junction. Scale bars: **(B–D)** = 250 μm; **(E–G)** = 25 μm.

Mouse tissue sections or explants were stained as described previously (Fueshko and Wray, 1994). In brief, explants and cryosectioned (16 μm) fresh-frozen tissue (mouse embryos, postnatal heads, and adult brains) were fixed with 4% formaldehyde (1 h), incubated in 10% NGS/0.3% Triton X-100 (1 h), placed in primary antibody (overnight at 4°C), incubated in biotinylated-goat anti-rabbit or anti-mouse (1 h; 1:500 in 0.3% Triton X-100; Vector Laboratories, Burlingame, CA), and processed using a standard avidin–biotin–horseradish peroxidase/3′, 3-diaminobenzidine (DAB, Sigma) protocol. Antibody specificity was assessed by elimination of the primary antibody and inclusion of knockout (Figure 1D) and control tissue. Double–label ICC was performed using DAB (brown reaction) and SG substrate (Vector Laboratories; blue reaction) as described previously (Giacobini et al., 2004). For double-immunofluorescence experiments, primary antisera were diluted as follows: GnRH (1:1000), p75NGFR (1:1500), nestin (1:3000), S100 (1:1000–4000), and GFAP (1:5). Alexa-Fluor 488 secondary antibody or with Avidin Alexa Fluor 488 (1:1000, Molecular Probes, Eugene, OR) was used to visualize the first antigen–antibody complex, followed by blocking with anti-rabbit Fab fragment (80 μg/ml, 1 h, Jackson ImmunoResearch, West Grove, PA; Kramer et al., 2000a,b), fixation (4% formalin, 20 min), and incubation with second primary antibody, which was visualized with goat anti-rabbit CY-3 (1:1200, Jackson ImmunoResearch, West Grove, PA). Controls for double staining revealed no significant cross-reactivity (data not shown).

### *in vivo* analysis of p75NGFR KO mice

#### GnRH system

Serial sections were made from E16.5 mice (*N* = 5) and adult brains (*N* = 6–7). For both stages, 2–3 series were stained for GnRH. After staining, the number of GnRH cells (stained soma in section) was counted [nose and nasal forebrain junction (E16.5) and brain (E16.5 and adult); *N* ≥ 3 for each group]. Within the brain, the relative position of GnRH cells was determined. At E16.5 (cut parasagitally), a diagonal line from the anterior commissure (AC) to optic nerve/optic chiasm was used as a rostral vs. caudal boundary (see Figure 3A schematic). In the adult brain (cut coronally), three anatomical groupings were used: rostral (anterior to the crossing of the AC), medial (from AC crossing to beginning of supraoptic nucleus) and caudal (supraoptic nucleus to median eminence). Counts were multiplied by the total number of series and presented as mean ± s.e.m. Contingency tables were constructed for each age and the statistical test for independence, Chi square (χ^2^; Statview Software, Abacus Concepts, Inc., Berkely CA), was performed. This analysis indicates whether the observed differences signify real differences among populations or if there are differences that one might obtain in a similar population. Using this analysis, the expected GnRH cell number was calculated based on the hypothesis of independence, and the expected GnRH cell number compared to the counted cell numbers. In addition, subsequent cell χ^2^ analysis determined the relative contribution of each region to the differences observed. A stringent *p*-value of 0.0001 was chosen for significance because this test is based on GnRH cell number (large *N*).

Photomicrographs of GnRH neurons (*N* = 27–29 cells per genotype) from E16.5 and adult brains were taken and the diameter, perimeter, and area of cells determined using NIH imageJ. Data are presented as mean ± s.e.m. Student's *t*-test was performed on the resulting values.

#### Olfactory axons and OECs

Olfactory axons were identified by staining for peripherin (Wray et al., 1994), PSA-NCAM (Calof and Chikaraishi, 1989; Miragall et al., 1989; Yoshida et al., 1999), and GAD67 (Lee et al., 2008). GFAP, S100, and Sox10 were used as markers for OECs (Forni et al., 2011). Photomicrographs (3–5 photos/series/animal) of the vomeronasal organ and the nasal forebrain junction (NFJ) were taken and optical density determined using NIH ImageJ. Images were converted to black and white and threshold applied (auto ranging between 0 [lower limit] and 140 [upper limit]) to highlight stained fibers (see Figures 4E′,F′). The area of highlighted structures was measured and non-specific artifacts (such as bone) subtracted from the total measurement to obtain the area of stained fibers. Fiber density is reported as mean ± s.e.m. Statistical analysis was performed using a One-Way ANOVA. A *p*-value of 0.001 was chosen for significance.

### Perturbation of p75NGFR signaling *in vitro* using p75NGFR blocker

#### Acute treatment

Explants (3div or 6div, *N* = 3 for each stage) were placed in a temperature-regulated chamber 28°C with 5% CO_2_ and 5% humidity using a Live Cell Chamber (Pathology Devices, Inc., MD) mounted on a Nikon inverted microscope equipped with a Retiga CCD camera. Fields containing GnRH cells were selected based on cell morphology, location, and association with fibers (Fueshko and Wray, 1994). Time-lapse microscopy was conducted with media only (SFM, *t* = 0–40 min) to establish baseline cell movement; this was followed by infusion for another 40 min of SFM + anti-p75NGFR (Chemicon, 1:6000) or of SFM + IgG (1:6000). Antibody dilution and protein concentration were chosen from the literature (Zhang et al., 2009) and modified on the basis of preliminary experimental tests in explants [i.e., concentration of antibody which did not disrupt overall explant appearance]. Solutions were infused at a rate of 1 ml/min with a peristaltic pump. Media recycling was performed during each period. Images were acquired at 2 min intervals and time-lapse recordings of cell movement were generated using iVision-Mac software (BioVision Technologies, PA). Cell movement was measured using NIH imageJ and the data were analyzed by paired *t*-test. The results are reported as mean distance traveled by cells ± s.e.m. The identity of the recorded cells (GnRH-positive) was verified *post-hoc* by immunocytochemistry.

#### Chronic treatment

Explants were maintained in SFM containing either a blocking p75NGFR antibody (Chemicon, 1:6000) or rabbit IgG (1:6000, control group) from 3 to 6div (*N* = 3 for each stage). At 6div, explants were processed for single- or double-label ICC. GnRH cell number and morphology, olfactory axon complexity, and outgrowth as well as morphology of OECs were analyzed. A second group of nasal explants was treated with human recombinant NT3 (5 ng/ml, Millipore, Billerica, MA), or human recombinant NT3 + p75NGFR antibody and GnRH cell counts and morphology quantified.

GnRH-immunopositive cells were counted on the main tissue mass (IN) and in the periphery of the explant (OUT) (Fueshko et al., 1998; Giacobini et al., 2004). The main tissue mass contains the nasal pit, nasal epithelial region, mesenchyme, and nasal midline cartilage (see Figure 8A). The periphery refers to the area surrounding the main tissue mass into which cells have spread and/or migrated. The cell number in the main tissue mass plus periphery, equal the total number of GnRH cells in the explant. Data are presented as mean ± s.e.m. Statistical analysis was performed using a One-Way ANOVA. A *p*-value of 0.001 was chosen for significance. GnRH cell perimeter, diameter and area were determined and statistical analysis was performed as above.

Morphological measurements of OECs were obtained (NIH ImageJ) in two different regions of the explant periphery—proximal and distal to the tissue cell mass in anti-p75NGFR (*N* = 3) and IgG (*N* = 3) groups. After changes in OEC morphology were noted, actin elements within S100 positive cells were examined. Optical density measurements of olfactory axons were also performed on explants treated with anti-p75NGFR and IgG and immunostained for peripherin. Photomicrographs (2–3) of each explant were taken in periphery (*N* = 3 explants/group; 8 micrographs/group). Optical densities of olfactory fibers were measured and statistical analysis done as above. Values are given as the mean optical density ± s.e.m. A mean distance of fiber outgrowth was also obtained.

## Results

### P75NGFR expression in GnRH neurons and OECs

GnRH neurons migrate from the nasal placode into the forebrain apposed to a subset of olfactory axons (Figures 1A,B). In mouse, this migration mainly occurs between E12.5–E17.5 (Wray et al., 1989). Characterization of p75NGFR in the nasal region during this period revealed immunoreactive elements in track-like structures emerging from the VNO (Figure 1C). Double-labeling for GnRH and p75NGFR indicated that many GnRH neurons were p75NGFR-positive, whether adjacent to the VNO (Figure 1E) or at the NFJ (Figure 1F). Once within the brain (E14.5), it was difficult to determine whether GnRH neurons maintained p75NGFR expression because of the high presence of this receptor in other CNS cells (data not shown). However, in the adult, p75NGFR protein was not observed in GnRH neurons (Figure 1G, arrowhead) although p75NGFR-positive cells were detected in nearby brain regions (Figure 1G, arrows).

**Figure 2:**
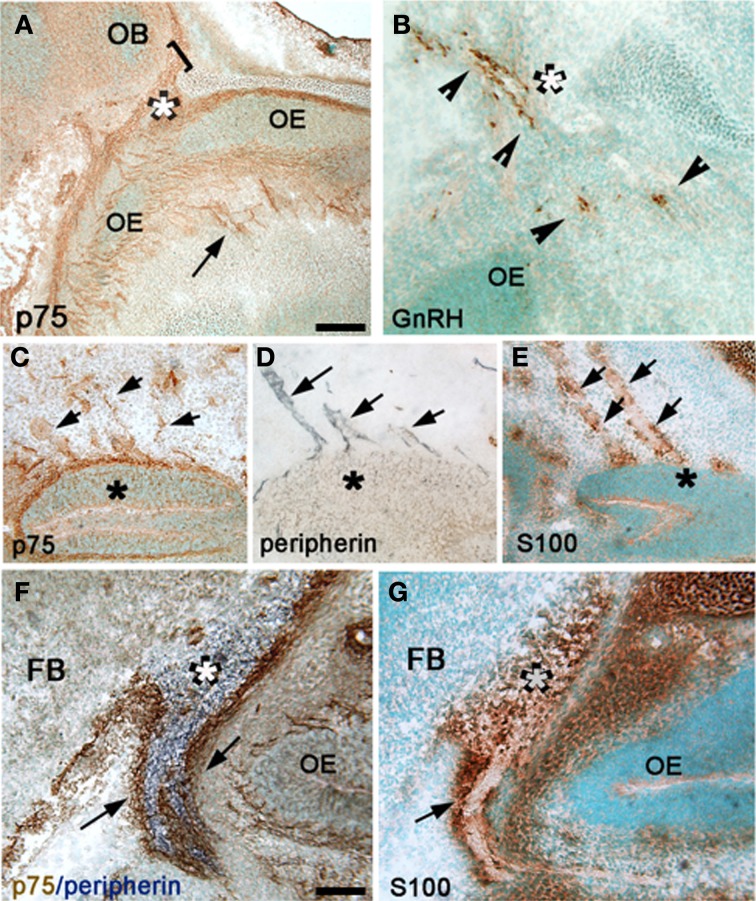
**P75NGFR cells are associated with the developing olfactory system—OECs. (A,B)** Comparison of p75NGFR staining (**A**, p75) and GnRH staining **(B)** indicated that more structures express p75NGFR than just the GnRH cells. p75NGFR staining **(A)** highlighted track-like structures in the nose (arrow) around the OE and crossing through the nasal mesenchyme, as well as at the nasal/forebrain junction (white asterisk) extending into the developing olfactory bulb (OB, bracket). At this development stage, GnRH neurons (**B**, arrowheads) cross the nasal/forebrain junction (white asterisk) and turn caudal into the FB. **(C–E)** Sagittal sections of the nasal region stained for p75NGFR **(C)**, peripherin **(D)**, and S100 **(E)** indicate that both p75NGFR- and S100-positive elements are distributed along olfactory axons (expressing peripherin) extending from the VNO (asterisk). At this developmental stage S100 is a marker for OECs. **(F)** Double immunocytochemistry for p75NGFR (brown) and peripherin (blue) revealed p75NGFR immunopositive cells (arrows) at the nasal forebrain junction (white asterisk) that apposed peripherin positive olfactory axons (blue). Note p75NGFR and peripherin did not colocalize in olfactory fibers. **(G)** Serial consecutive section to that in **(F)**, immunostained for S100 (brown, arrow) showing robust labeling in a pattern similar to that of p75NGFR (compare **F** and **G**). Scale bars: **(A–E)** = 250 μm; (**F,G)** = 50 μm.

Between E12.5 and E17.5, p75NGFR immunoreactivity in the nasal region was also detected in structures other than GnRH neurons (compare Figures 2A,B, see Figures 1E,F). These p75NGFR immmunoreactive-elements coincided with the migratory pathway taken by GnRH neurons, detectable from the VNO (Figure 2C) through the NFJ (Figure 2A) and were associated with the nerve layer entering the olfactory bulbs (Figure 2A, bracket). Immunocytochemistry for p75NGFR (Figures 2C,F) and peripherin (Figures 2D,F) confirmed that p75NGFR positive-elements were distributed along the axons that emerged from the VNO (Figure 2C) and targeted the olfactory bulb (Figure 2F). The pattern of S100 staining (Figures 2E,G), a glial marker expressed by OECs, resembled that of p75NGFR (compare 2C and E, 2F and G). These data are consistent with OECs, at this early stage, expressing p75NGFR. The spatiotemporal expression of p75NGFR suggested that signals via this receptor could play a role in migration of GnRH neurons in nasal regions and/or olfactory system development. To begin to address this issue, p75NGFR^−/−^ mice were examined.

### Lack of p75NGFR affects morphology and distribution of GnRH neurons

**Figure 3:**
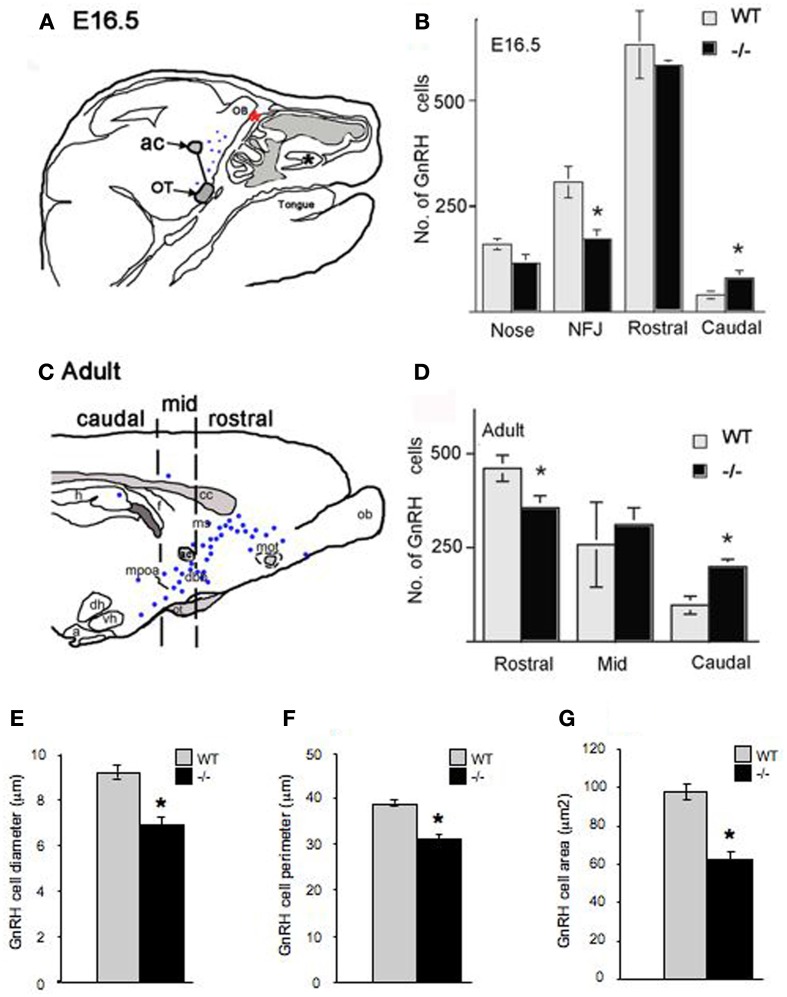
**Altered morphology and distribution of GnRH neurons in p75NGFRmutant (-/-) embryos**. At E16.5, the majority of GnRH neurons have migrated into the forebrain associated with a subpopulation of olfactory axons. **(A)** Camera lucida of E16.5 sagittal head showing nasal forebrain junction (red asterisk) and location of anterior commissure (ac) and optic tract (OT) that were used as markers, from which a diagonal line was drawn separating rostral from caudal GnRH cell population. GnRH cells were found in the nasal area [between the VNO (black asterisk) and nasal forebrain junction (NFJ, red asterisk)], at the NFJ, and within the forebrain (black dots). **(B)** Histogram showing distribution of GnRH cells in the nasal and brain regions at E16.5, a decrease in the number of GnRH cells in the NFJ with a corresponding increase in the caudal cell population is observed in -/- mice. **(C)** Camera lucida of adult brain showing location of markers for rostral = before anterior commissure (ac), medial = between ac and before suporaoptic nucleus, Blue dots = location of GnRH cells, and caudal = from supraoptic nucleus to caudal brain areas. **(D)** In the adult, a decrease in the GnRH population within the rostral brain region with corresponding increase in caudal brain was observed. Measurements of GnRH cell diameter **(E)**, perimeter **(F)** and area **(G)** revealed a significant reduction in all three parameters in p75NGFR^−/−^embryos. ^*^p < 0.001.

Between E14.5 and E17.5, GnRH neurons (numbering 800–1000 cells) were found in the nasal area, at the NFJ and within the forebrain (Figure 3A). GnRH cell number in p75NGFR^−/−^ mice was similar to controls (E16.5: WT = 1138 ± 35, p75NGFR^−/−^ = 950 ± 57, *p* > 0.05). However, examination of the spatial location of GnRH neurons showed a shift in the cell distribution; in particular, GnRH cell number decreased by ~50% at the NFJ (Figure 3B, WT = 307 ± 37, KO = 172 ± 22, *X*^2^ = 149.7 *p* < 0.0001) and increased by ~50% in the caudal brain region in -/- mice (WT = 40 ± 9, KO = 80 ± 18). Notably, the absolute change in number of cells at the NFJ (−135) did not equal the number in caudal regions (+40). Since the total GnRH cell number remained similar between genotypes, the difference in absolute values likely represents cells still in transit between the NFJ and caudal hypothalamus at E16.5.

Also apparent at this age was a change in the morphology of GnRH cells in the brain of mutant mice. The measurements of GnRH cell diameter, perimeter and area revealed a significant reduction in all three parameters in p75NGFR^−/−^ embryos [diameter, μm: WT = 9.18 ± 0.29, KO = 6.86 ± 0.35; perimeter, μm: WT = 38.68 ± 0.73, KO = 30.83 ± 1.09; area, μm^2^: WT = 97.75 ± 4.00, KO = 62.21 ± 3.95; *p* < 0.001 for each, Figures 3E–G]. To determine whether deletion of the p75NGFR gene altered the phenotype of other hypothalamic peptidergic neurons, the magnocellular system was evaluated using an antibody that stains both vasopressin and oxytocin cells. A robust signal, in both genotypes, was detected in fibers in the median eminence and in cell groups corresponding to the supraoptic and paraventricular nucleus. No change in cell morphology was found (data not shown). Thus, the altered phenotype observed in GnRH cells in p75NGFR^−/−^ at E16.5 mice was not a general effect.

To determine if the changes noted in the GnRH system at E16.5 in mutant mice were maintained, adult animals were examined (Figures 3C,D). Consistent with the data at E16.5, (1) the total number of GnRH cells was similar in p75NGFR^−/−^ and WT adult mice (WT = 815 ± 123; KO = 867 ± 15, *p* > 0.05) and (2) more cells were located in caudal regions. There was a 33% decrease in the number of GnRH cells in rostral brain regions (Figure 3D, WT = 461 ± 35, KO = 356 ± 32, *X*^2^ = 156.6 *p* < 0.0001); and a 50% increase in cells in the caudal region in ^−/−^mice (WT = 97 ± 24, KO = 199 ± 20). Thus, the shift in the location of GnRH cells detected at E16.5 was also found in adults. However, in adult brain, the absolute change in number of GnRH cells was similar (−105 rostrally vs. + 102 caudally). In contrast to E16.5^−/−^ mice, no differences in shape and size of GnRH cells were noted in adult mice (data not shown). These data indicate that altered p75NGFR signaling *in vivo* results in (1) a transient change in GnRH cell morphology and (2) altered migration of GnRH cells in the forebrain.

### P75NGFR alters density of olfactory fibers and OECs

**Figure 4:**
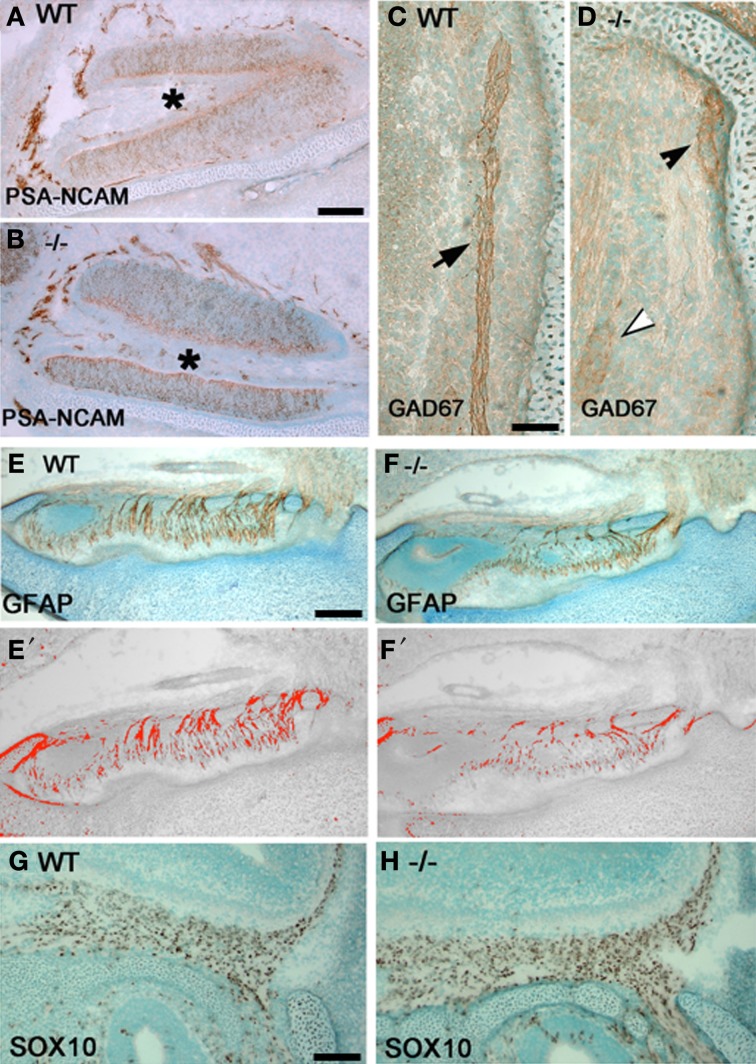
**Olfactory fiber and OECs changes in p75NGFR^−/−^ mice**. Sagittal sections close to midline of a p75NGFR^−/−^ **(B,D,F,H)** embryo and WT littermates **(A,C,E,G)** showing an area of VNO **(A,B)** and nasal forebrain junction **(C,D,E–H)**. Sections were immunostained for markers of olfactory axons—PSA-NCAM **(A,B)** and GAD67 **(C,D)**, and for OECs—GFAP **(E,F)** and SOX10 **(G,H)**. **(E′–F′)** are representative transformed images corresponding to **(E)** and **(F)**, respectively, and were used for measurement of olfactory fiber density; similarly transformed images (not shown) were used in optical density measurement for Sox10 positive cells in **(G)** and **(H)**. Optical density values from structures other than the fibers were subtracted from the total measurements. **(A,B)** PSA-NCAM-immuostained fibers around the VNO have similar pattern and intensity between WT **(A)** and **-/- (B)** animals. **(C,D)** GAD67 staining intensity in WT control animals is strong (**C**, brown, arrow); whereas, variable staining pattern observed in^−/−^ mice **(D)**, from weak (white arrowhead), moderate (black arrowhead) to absent. **(E,F)** Optical density measurement of GFAP-positive fibers in NFJ showed significant reduction in fiber density of p75^−/−^ mice compared to its WT littermates. **(G,H)** Optical density measurement of OECs in the NFJ showed increase in staining intensity in **-/-** mice but cell counts were similar with WT animals. Scale bars: 250 μm.

To determine if changes in GnRH neuronal migration correlated with changes in olfactory system development, olfactory fibers and OECs in two regions, the NFJ and around the VNO, in p75NGFR^−/−^ and WT mice at E16.5 were examined (Figure 4). Evaluation of olfactory fibers was performed using PSA-NCAM, peripherin, and GAD67. PSA-NCAM and peripherin immunostained a large number of olfactory fiber bundles in the nasal region, while a fraction of the fiber bundles is GAD67-immunoreactive. The PSA-NCAM- (Figures 4A,B) and peripherin-positive (not shown) olfactory fibers showed similar patterns in WT and KO, whereas GAD67-immunostained fibers differed (Figures 4C,D). Compared to the strong immunostaining detected in all WT embryos (Figure 4A), moderate to absent GAD67-immunopositive fibers was found in mutant mice (Figure 4D).

Evaluation of OECs in the VNO and NFJ was performed using antibodies to GFAP and SOX10. In the VNO, the density of OEC somas and processes were similar in WT and -/- p75NGFR embryos (data not shown). However, within the NFJ, optical density measurements of GFAP-positive fibers revealed a 33% decrease in mutant embryos compared to WT (Figures 4E–F′; WT = 155.6 ± 0.12; KO = 51.2 ± 15.9, *p* < 0.005). In contrast, optical density measurements of Sox10-positive OEC soma increased in mutant mice [Figures 4G,H; WT = (2.9 ± 0.6) × 10^5^, KO = (7.7 ± 0.6)× 10^5^, *p* < 0.0001] but cell counts in representative regions of the NFJ were similar. These data suggest that the increase in optical density was due to an increase in SOX-10 expression in OECs and not an increase in the number of OECs. To further clarify the mechanism by which signaling via the p75NGFR altered GnRH cell migration and/or olfactory fiber and OEC development, functional studies were performed in nasal explants.

### Functional analysis of p75NGFR in nasal explants

**Figure 5:**
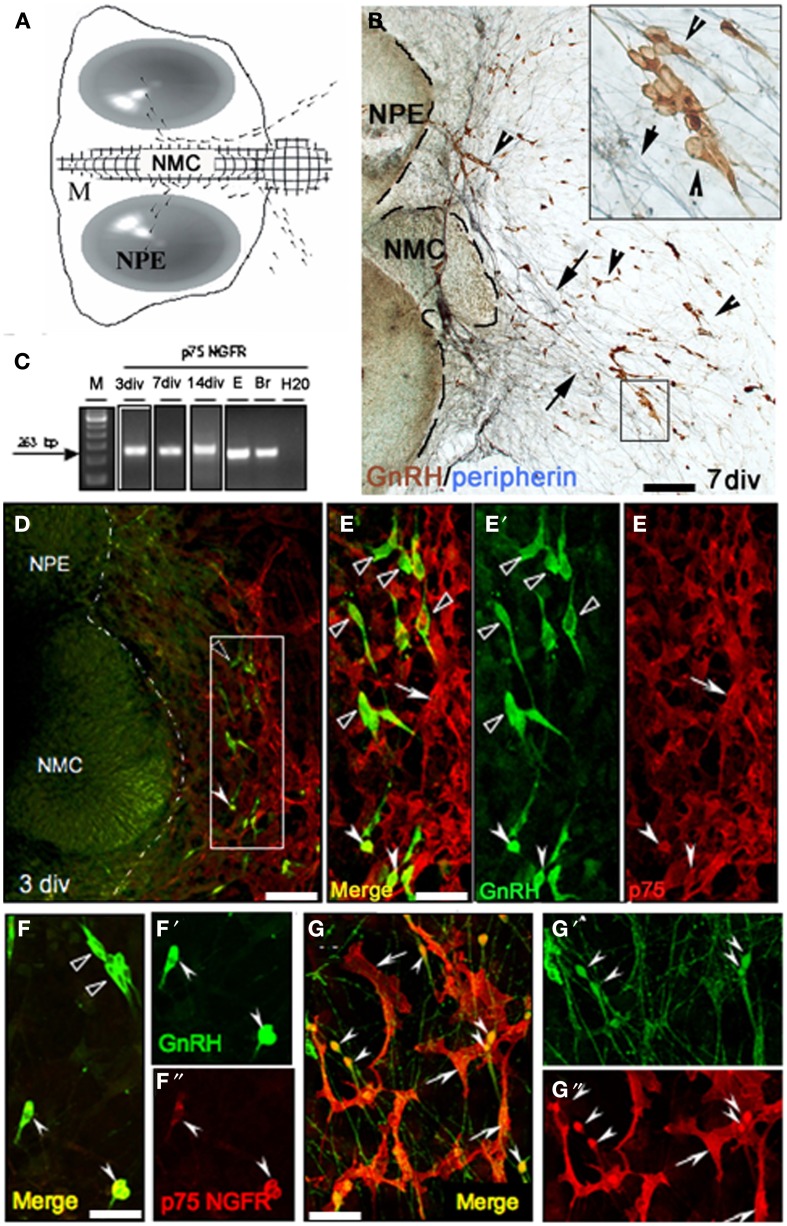
**P75NGFR expression in nasal explants mimics expression *in vivo*. (A)** Schematic of nasal explant showing the migration of GnRH neurons (black dots) across the main tissue mass (M) from the nasal pit/epithelial region (NPE) to the nasal midline cartilage (NMC), and then into the periphery of the explant. **(B)** Double immunocytochemistry showing GnRH neurons (brown, arrowheads) and peripherin fibers (blue, arrows) on a 7 div nasal explant. At this stage the majority of GnRH neurons have migrated off the main tissue mass into the periphery along peripherin-immunoreactive axons (see insert). **(C)** Gel documentation of PCR products using specific primers for p75NGFR on single GnRH cell at 3, 7, 14 div, E17.5 head **(E)** and adult brain (Br). A band of expected size (263 bp) was detected in GnRH cells in nasal explants at all 3 ages. No band was detected in water. **(D,E)** Double immunofluorescence using antibodies against GnRH (green) and p75NGFR (red) at 3div showed that the majority of GnRH neurons localized proximal to the main tissue mass did not express p75NGFR (**E–E″** = high magnification of box area in **D**, black arrowheads); however, p75NGFR immunoreactivity was detected in some GnRH neurons (**E–E″**, white arrowheads) and in a multipolar phenotype also out in the periphery of the explant (arrows, **E–E′**, dashed line in **D** indicates the border between the tissue mass and the periphery). **(F–G″)** At 7 div GnRH neurons (green) in the proximal aspect of the periphery of the explant (close to the main tissue mass, **F–F″**) were either p75NGFR negative (black arrowheads), or showed co-expression of p75NGFR protein (white arrowheads). Distally **(G–G″)**, the expression of p75NGFR in migrating GnRH neurons increased as function of distance from the tissue mass/into the periphery (arrowheads). Note, p75NGFR multipolar positive elements (arrows) were closely associated with both soma and fibers of GnRH neurons. Scale bars: **(B,D)** = 250 μm; **(E–G″)** = 50 μm.

GnRH cells maintained in nasal explants (Figures 5A,B) exhibit many characteristics similar to those observed *in vivo* (Fueshko and Wray, 1994). In explants, GnRH neurons (Figure 5B, brown) migrate from the nasal pit epithelia (NPE) into the periphery apposed to peripherin-positive olfactory fibers (Figure 5B, blue). GnRH cells move to the edge of the main tissue mass (Figure 5B dotted line) after 1–3 days *in vitro* (div) and then to more distal sites in the periphery from 3 to 7 div.

Prior to initiating p75NGFR perturbation studies, the expression of p75NGFR in cells was characterized in nasal explants. PCR for p75NGFR was performed on cDNAs from single GnRH cells previously generated (Kramer et al., 2000a; Giacobini et al., 2007; Constantin et al., 2009). A band of the appropriate size (263 bp) was detected Figure 5C; at 3–4div (~33%, *n* = 7/21) and 7div (~33%, *n* = 7/22). Double-immunofluorescence confirmed expression of p75NGFR in GnRH neurons. At 3div, GnRH neurons have begun to migrate into the periphery, but are still proximal to the main tissue mass (Figures 5D,E–E″ = high magnification). At this stage, a subpopulation of GnRH neurons was p75NGFR positive (Figures 5E,E″, white solid arrowheads). At 7 div the majority of GnRH neurons have migrated off the main tissue mass (Figures 5F–G″). p75NGFR was still detected in a subpopulation of GnRH neurons proximal to the main tissue mass (Figures 5F–F″, white solid arrowheads). However, more p75NGFR-positive/GnRH cells were present, with many intensely double-labeled cells now detected in the distal area of the explant periphery (Figures 5G–G″, arrowheads).

**Figure 6:**
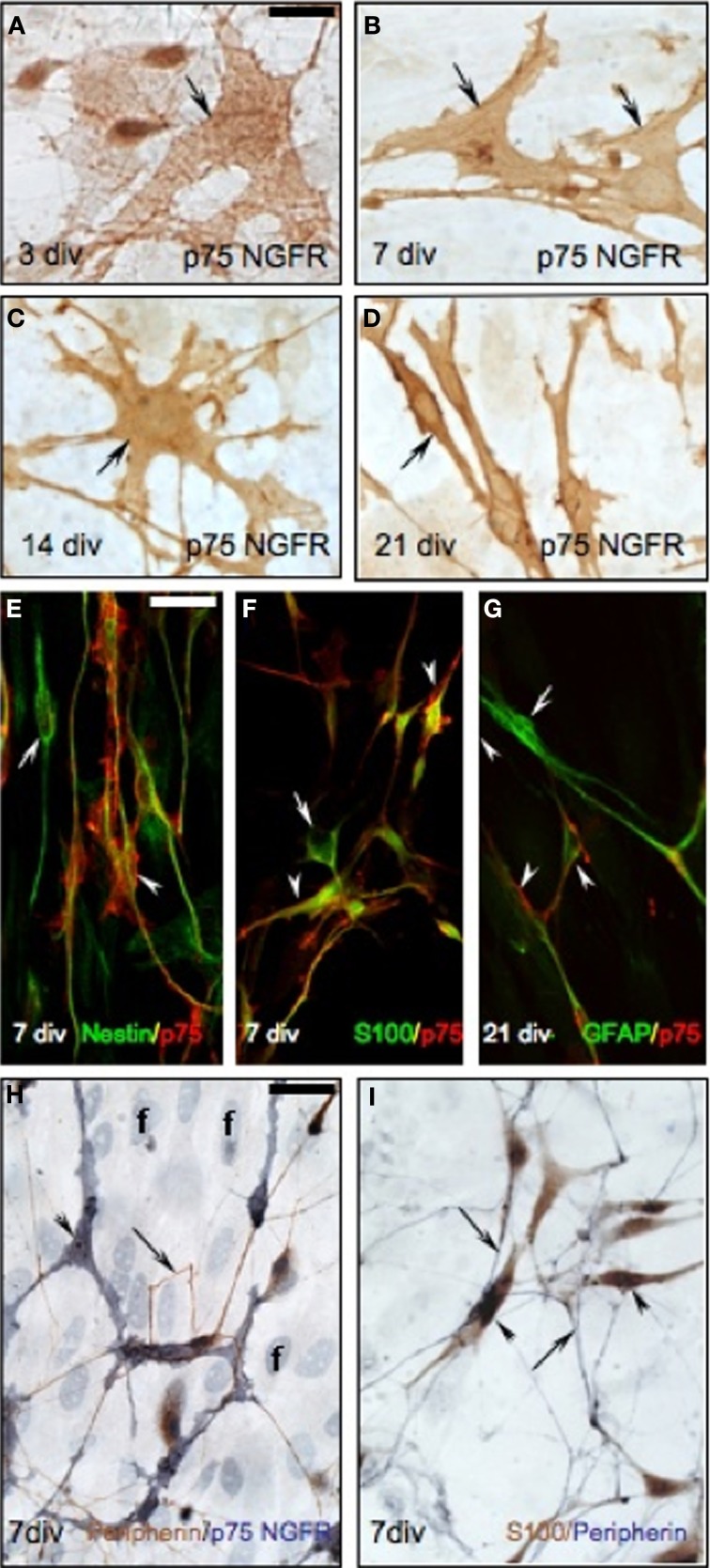
**Characterization of p75NGFR positive OECs in nasal explants. (A–D)** p75NGFR-immunoreactive cells (brown, arrows) in the periphery of nasal explant undergo morphological changes, increasing process extensions between 3 **(A)**, 7 **(B)**, and 14 **(C)** div, and changing from multipolar to bipolar between 14 **(C)** and 21 **(D)** div. **(E)** Double-immunofluorescence using nestin (green) and p75NGFR (red) antibodies at 7 div showed a subpopulation of p75NGFR-positive multipolar cells (arrowhead) coexpressed nestin, an early neural marker. Some nestin-positive/p75NGFR-negative cells were also evident (arrow). **(F)** Double-label immunofluorescence colocalized S100 (OEC marker, green) and p75NGFR (red) at 7 div (arrowheads). Some S100-positive/p75NGFR-negative elements (**F**, arrow) were also detected in the periphery of the explant. **(G)** Double-label fluorescence at 21 div showed a subpopulation of p75NGFR-positive cells (red) expressed GFAP (OEC marker, arrowhead). Some GFAP-positive/p75NGFR- negative cells were also present (**G**, arrow). Note, in all double- immunofluorescence images **(E–G)** p75NGFR reactivity appeared localized to the surface of the cell membrane. **(H)** Double-immunostaining for peripherin (brown, arrows) and p75NGFR (blue, arrowheads) at 7 div. In the periphery of the explant, peripherin-positive axons (arrows) were closely associated with p75NGFR multipolar-positive OECs cells (arrowheads). In fact, the olfactory axons appeared to preferentially grow to p75NGFR OECs. **(I)** Double-immunocytochemistry against the OEC marker, S100 (brown, arrowheads) and peripherin (blue, arrows) at 7 div showed a similar pattern to that in panel **(H)**. Scale bars: = 250 μm.

As detected in the nasal region *in vivo*, P75NGFR-positive/non-GnRH cells were also present in the periphery of the explants (3–21 div, Figures 5D–G), exclusively on the side from which the GnRH cells and olfactory axons exited the explant. These cells were multipolar (exhibited several short extensions), larger than GnRH cells but in close apposition to both the soma and processes of GnRH neurons (Figures 5D–G″). The morphology of these p75NGFR-positive cells drastically changed as a function of the time: at 3div, they were large, flat cells with few well-defined processes (Figure 6A, arrow); by 7 div, the cells had become more triangular (Figure 6B, arrows); by 14div, the cells were star-shaped with numerous short processes (Figure 6C, arrow); and by 21div they were elongated becoming spindle-shaped often with a central defined nucleus and reduced processes (Figure 6D, arrow). Fibroblasts were not p75NGFR positive (Figure 6H, examples labeled f). Thus, it appeared that the majority of p75NGFR+/GnRH− cells in the explants, as in nasal regions *in vivo* were OECs. This was confirmed using cell differentiation markers including nestin, a marker of neural progenitors; S100, a marker for OECs; and GFAP, a marker for astrocytes and OECs. At 3 and 7div, these cells co-expressed nestin (Figure 6E, arrowhead) but not GFAP (data not shown). Between 7 and 14 div, nestin labeling decreased. A large proportion of these p75NGFR-positive cells expressed S100 protein by 7 div (Figure 6F, arrowheads) and GFAP by 21 (Figure 6G, arrowheads). These cells were associated with olfactory axons (Figures 6H,I). Thus, as *in vivo*, OECs in nasal explants expressed p75NGFR, S100, and GFAP, associated with GnRH cells and olfactory axons and underwent developmental changes in gene expression indicative of cell maturation. Since the expression of p75NGFR in explants mimicked that characterized *in vivo*, perturbation experiments were performed. Two temporal windows were used to examine the role of p75NGFR: 3 div, a time characterized by massive olfactory axonal growth and GnRH neuronal migration into the periphery; and 6 div, a time of little movement and outgrowth.

#### Acute inhibition of p75NGFR alters migration rate of GnRH neurons

To address whether the altered distribution of GnRH cells *in vivo* was due to altered cell movement, GnRH cell migration was monitored *in situ* during acute treatment with a p75NGFR antibody known to block receptor function (Zhang et al., 2009). Explants at 3 or 6div were exposed to fresh serum free media (SFM, 40 min) followed by SFM + anti-p75NGFR (treated, 40 min) or SFM + IgG (control, 40 min). The mean path distance of GnRH cells was compared prior to and after exposure to anti-p75NGFR or IgG. In general, the rate of migration of GnRH cells (in SFM) at 3 div was faster than at 6div. This is consistent with data obtained by Casoni et al. (2012). Mean path distance traveled by GnRH cells exposed to SFM + IgG was similar to that recorded in SFM at each age (3 div: SFM = 30.3 ± 1.26 mm/min, SFM + IgG = 29.4 ± 1.6 mm/min, *n* = 32, *N* = 2, *p* > 0.05; 6 div: SFM = 14.1 ± 1.1 mm/min, SFM + IgG = 15.6 ± 0.9 mm/min, *n* = 46, *N* = 2, *p* > 0.05). Treatment of explants with anti-p75NGFR at 3div did not affect mean path distance traveled by GnRH cells (SFM = 30.0 ± 1.7 mm/min, *n* = 39, *N* = 2; SFM + anti-p75 = 27.3 ± 1.6 mm/min, *n* = 39, *N* = 2, *p* > 0.05). In contrast, treatment at 6 div significantly increased the mean path distance (SFM = 14.1 ± 1.1 mm/min, *n* = 46, *N* = 2, *p* < 0.05; SFM + anti-p75 = 16.2 ± 0.7 mm/min, *n* = 75, *N* = 3, *p* < 0.05). These results show that p75NGFR can modulate the rate of GnRH migration at later stages *in vitro* when relatively more GnRH cells express p75NGFR (Figures 5F–G″) and migration rate is slowing.

#### Chronic inhibition of p75NGFR affects morphology of OECs, fasciculation of olfactory fibers and morphology and number of GnRH neurons

**Figure 7:**
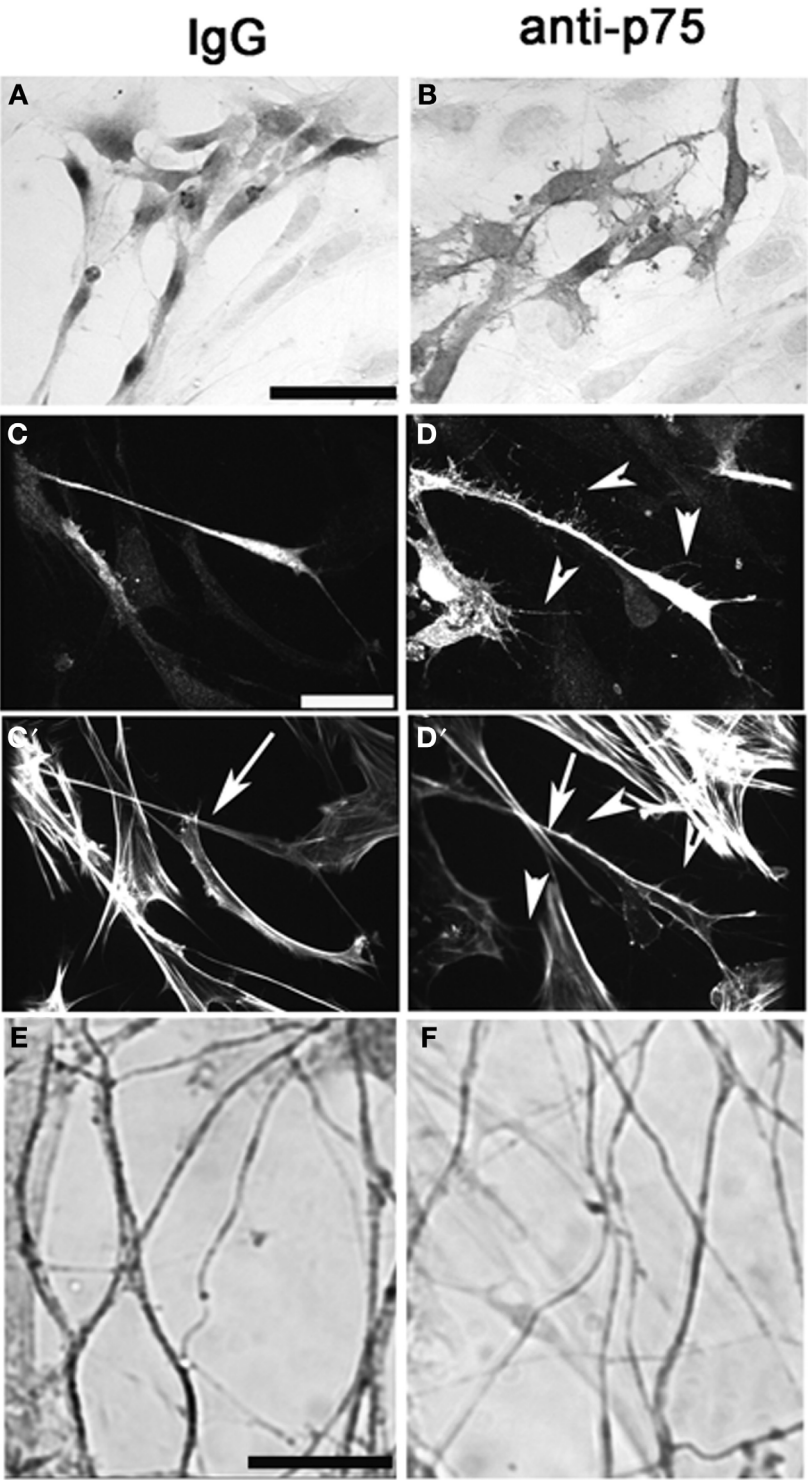
**Perturbation of p75NGFR signaling *in vitro* alters morphology of OECs and causes defasciculation of olfactory fibers**. Nasal explants at 6div following treatment with p75NGFR blocking antibody (1:6000, **B,D,F**) and with rabbit IgG mock, 1:6000, **(A,C)**, Explants were immunocytochemically-stained for S-100 **(A,B)**, S-100 and Phalloidin **(C,D)** or peripherin **(E,F)**. **(A–D)** Disruption of p75NGFR signaling alters morphology of OECs The morphology of S100-positive cells located distal to the explant's tissue mass were larger, flatter, and exhibit more processes and neurites **(B,D)** as compared to control group **(A,C)**. Staining for actin (phalloidin) revealed that these processes had sparse actin filaments (**D′**, arrowheads). OECs in the treated group also appeared to have denser cortical actin along the axis of the cell body (**C′** vs. **D′**, arrow). Finally, disruption of p75NGFR increased olfactory fiber branching and decreased the optical density of peripherin-positive fibers, suggesting defasciculation had occurred. Scale bars: = 250 μm.

Explants were treated with anti-p75NGFR or IgG at 3div and OECs, olfactory axons and GnRH neurons analyzed at 6 div. S100-positive cells located proximal to the tissue mass showed no differences in size and shape between the groups (mean area: mock = 173.0 ± 11.0 μm^2^, anti-p75NGFR = 152.0 ± 12.7 μm^2^, *N* = 3 for each group, *p* > 0.05). In contrast, in the anti-p75NGFR-treated group, S100-positive cells in the more distal region were relatively flat, large, and had more processes and tiny lamellapodia and filopodia compared to those in the IgG-treated control group (Figures 7A,B; Area: mock = 286.0 ± 19.8 μm^2^, anti-p75NGFR = 443.0 ± 26.0 μm^2^, *N* = 3 for each group, *p* < 0.0001). Staining for S100 and actin (phalloidin, Figures 7C–D′) revealed that the distal OECs in the anti-p75NGFR group were densely stained S100-positive cells with multiple lamellapodia/filopodia (Figures 7C,D, arrowheads). These processes had sparse actin filaments (Figure 7D′, arrowheads). OECs in the treated group also appeared to have denser cortical actin along the axis of the cell body (Figures 7C′ vs. D′, arrow). No change in the overall length of peripherin-positive olfactory fibers was noted between groups. However, in anti-p75NGFR treated explants, an increase in single fiber branching and a decrease in optical density of peripherin-positive bundles were found (Figures 7E,F, Mock = 0.78 ± 0.057 um^2^; anti-p75NGFR = 0.69 ± 0.027 um^2^, *N* = 3 for each group, *p* < 0.001), suggesting de-fasciculation of olfactory fibers occurred with disruption of p75NGFR signaling.

In parallel to changes found in GnRH neurons in p75NGFR^−/−^ mice at E16.5, a significant decrease in the diameter, perimeter, and area of GnRH cells occurred in explants treated with anti-p75NGFR (Table 2). However, in contrast to p75NGFR KO mice, there was a significant reduction in the number of GnRH cells in treated explants. The reduction in GnRH cells occurred in the periphery of the explants and was not compensated for by an increase in GnRH cells on the main tissue mass [In: control = 32 ± 5, treated = 28 ± 5 (*p* > 0.05); Out: control = 118 ± 15, treated = 65 ± 7 (*p* < 0.05); Total cell number: control = 149 ± 16, treated = 93 ± 10 (*p* < 0.05); *N* = 3 for each group]. Caspase-3 staining was performed to determine whether a GnRH subpopulation was undergoing cell death. Explants were treated with anti-p75NGFR starting at 3div and then fixed at 12, 24, or 48 h after treatment. Although caspase-3-positive cells were identified in all explant groups, no GnRH-positive/caspase-3-positive cells were detected in treated or control groups (data not shown), nor did any other specific cell group (i.e., OECs) appear preferentially caspase-positive. Thus, the loss of ~38% of the GnRH cells in the periphery of the explant following anti-p75NGFR treatment does not appear to involve caspase-3-dependent apoptosis. Since no other phenotypic markers exist for GnRH cells, a loss of peptide production or delay in maturation/exit from the olfactory pit would also result in a decrease in GnRH cell number. Thus, it is presently unclear whether an indirect change occurred during chronic treatment *in vitro* and/or compensatory mechanisms are present *in vivo* that prevent GnRH cell death, decreased peptide production or delayed differentiation.

**Table 2:** **Summary of morphological analysis of GnRH neurons from treated nasal explants**.

**Parameters**	**Control (Rabbit IgG, 1:6000)**	**Anti-p75 NGFR (1:6000)**	**NT3 (5 ng/ml) + Rabbit IgG (1:6000)**	**NT3 (5 ng/ml) + Anti-p75 NGFR (1:6000)**
Diameter (μm)	7.88 ± 0.22	6.80 ± 0.36^a^^,^^b^	7.24 ± 0.26	6.64 ± 0.15^a^^,^^b^
Perimeter (μm)	42.14 ± 0.83	35.20 ±1.6^a^^,^^b^	42.92 ± 0.72	35.77 ± 0.68^a^^,^^b^
Area (μm^2^)	96.81 ± 2.53	74.20 ± 5.52^a^^,^^b^	108. 40 ± 3.80^a^	75.52 ± 2.73^a^^,^^b^

a Statistically different from control (p < 0.001)

b *Statistically different from NT3 + Rabbit IgG treated group (p < 0.001)*.

NGF, BDNF and NT3 and NT4 are known to interact with p75 NGFR and effect cell survival (Manadas et al., 2007). Thus, the presence of these ligands was evaluated in explants by PCR. NT3 transcript was present in all explants at 3 and 7div (*N* = 3 at each stage), while the presence of the other ligands were variable (NGF: 33% of total number of explants at 3div and none at 7div; BDNF: 17% of total number of explants at 3div and 7div; NT4,5: 33% of total number of explants at 3 and 7 div). Because these ligands are known to interact with tyrosine kinase receptors (TrkA, TrkB, and TrkC), the presence of these receptors in GnRH cells that expressed p75 NGFR were also evaluated. At 3.5–4.5 div (*n* = 19), 37% of GnRH cells were positive for p75NGFR; and TrkA, TrkB, and TrkC were present in 0, 43, and 86% of the p75NGFR-positive/GnRH-positive cells, respectively. At 7 div (*n* = 16), 44% of GnRH cells were positive for p75 NGFR; and TrkA, TrkB, and TrkC were present in 14, 57, and 71% of the p75 NGFR-1-positive/GnRH-positive cells, respectively.

**Figure 8:**
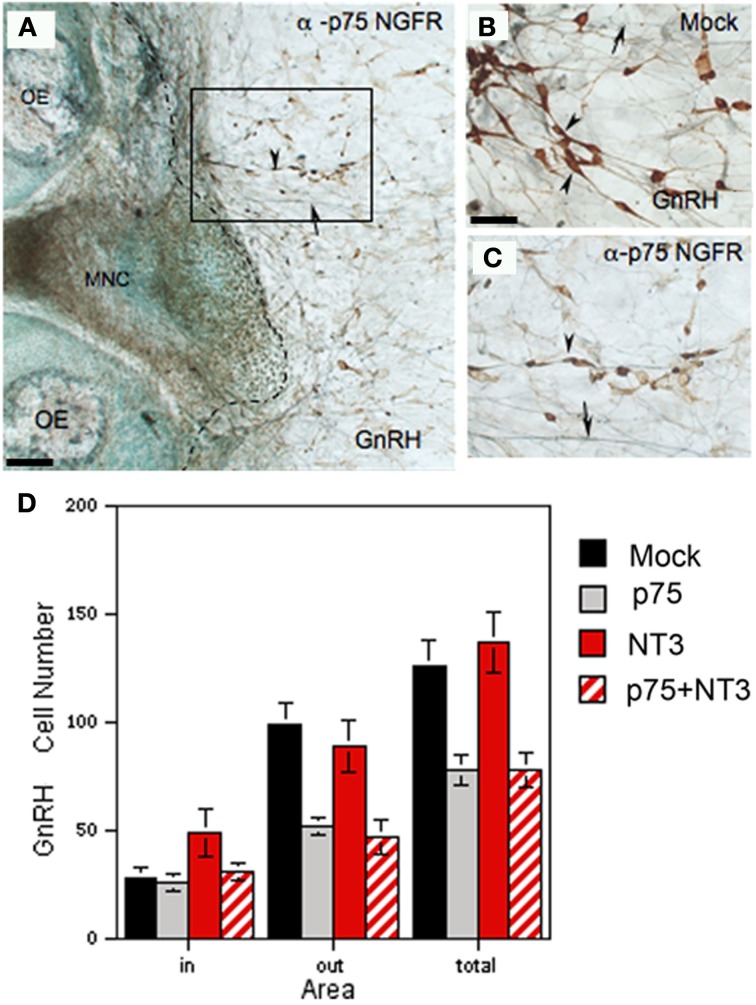
**Neutralization of endogenous p75NGFR decreases GnRH cell number. (A–C)** Nasal explants immunocytochemically stained for GnRH (brown, arrowheads) and peripherin (blue, arrows) at 6div after blocking antibody treatment (**A,C**; α-p75 NGFR) and in control condition (**B**, mock: rabbit IgG, 1:6000). Inhibition of p75 NGFR appeared to decrease the number of GnRH neurons in the periphery (**C**, arrowheads) as compared to the mock **(B)**, arrowheads, the dashed line in **(A)** indicates the border of the explant tissue mass. **(D)** Histogram showing the mean cell number of GnRH neurons detected in the tissue (in), in the periphery of explant (out) and the total number (total) in 4 treatment groups: control (mock, black), anti-p75NGFR (gray bars), NT-3 (red bars) and anti-p75NGFR+NT-3 (red hatched bars). Anti-p75NGFR ± NT-3 treatment significantly decreased the number of GnRH cells in the periphery (out) and total number of GnRH neurons (total) in comparison with the control and NT-3 treated groups (*p* < 0.001). No differences were found in the number of GnRH cells on the main tissue mass (in). Scale bars: **(A)** = 250 μm; **(B,C)** = 50 μm.

The presence of NT3 in all nasal explants and of TrkC in large population of GnRH cells suggested a possible role for this signaling system in GnRH cell survival (Vigers et al., 2003). Thus, pharmacological manipulations were performed exposing explants to NT3 (5 ng/ml) + rabbit IgG, NT3 + p75NGFR blocker, and p75NGFR blocker alone from 3 to 6div as described above. With respect to the morphology of GnRH neurons and number of GnRH cells in the explant, NT3 + rabbit IgG treatment (*N* = 8) produced results similar to controls except that a consistent increase in the area of GnRH neurons was observed (Table 2). In all aspects measured, GnRH cells in explants treated with NT3 + anti-p75NGFR (*N* = 5) showed changes similar to that found after treatment with p75NGFR blocker antibody alone (Table 2, Figure 8). Thus, addition of exogenous NT3 was unable to “rescue” the anti-p75NGFR effects on GnRH cells.

## Discussion

Signals transduced via p75NGFR have been implicated in a variety of functions including neuronal survival, axon growth, neurite outgrowth, and Schwann cell migration (Anton et al., 1994; Bergmann et al., 1997; Bentley and Lee, 2000). This report shows that GnRH neurons and OECs express p75NGFR at E12.5 in mouse and establishes a role for p75NGFR in modulating olfactory axon outgrowth, maturation of OECs, GnRH cell survival, and neuronal migration.

Prenatal expression of p75NGFR occurs in the trigeminal ganglion, dorsal root ganglion, and placode derived sensory ganglia (such as otic placode) as early as E11 in rat (Yan and Johnson, 1988). By E13, all peripheral nerves were positive due to p75NGFR in Schwann cells; and by E14, the nerve tract and glomeruli of the olfactory bulb were positive (Yan and Johnson, 1988). Further characterization by Gong et al. (1994), established that olfactory axons were p75NGFR negative but that the OECs were p75NGFR positive. During prenatal development, GnRH neurons arise from the olfactory placode and migrate across the nasal region in association with olfactory axons and OECs into the forebrain (Wray, 2001; Forni et al., 2011). Between E12.5 and E14.5 many embryonic GnRH neurons were found to co-localize p75NGFR protein, whereas in the adult, GnRH cells in the forebrain were p75NGFR-negative. The temporal expression of p75NGFR in GnRH neurons correlated with general expression of p75NGFR, being extensively expressed during early stages and down-regulated over development (Yan and Johnson, 1988; Gong et al., 1994). In addition to GnRH/p75NGFR-positive neurons, OECs were found to be p75NGFR-positive from E12.5 to E14.5. To investigate the involvement of p75NGFR in the developing olfactory system and GnRH system, *in vivo* and *in vitro* experiments were performed in p75NGF^−/−^ mice and nasal explants, respectively.

No change in GnRH cell number was found in p75NGFR^−/−^ mice. However, a shift in the relative position of GnRH cells was observed, with GnRH cells migrating further caudally toward the median eminence. Staining for neurophysin, a marker of magnocellular neurons in the periventricular nucleus and supraoptic nucleus and parvocellular cells in the suprachiasmatic nucleus (Wray et al., 1988) and NPY, a marker of cells in the arcuate nucleus (Hilal et al., 1996) revealed immunopositive cells in areas corresponding to that of wild type animals. The presence of these cellular markers in appropriate nuclei indicates that disruption of the p75NGFR gene did not affect general hypothalamic organization. In addition, both the internal and external zones of the median eminence were similar in p75NGFR mutant and control mice (data not shown). Thus, the shift in GnRH location in the brain was likely due to a change in migration of the GnRH cells. In fact, acute administration of p75NGFR blocker in nasal explants caused GnRH cells at 6div to migrate at a faster rate than controls. An increase in GnRH migration rate was not observed at 3div, when migration rate is maximal (Casoni et al., 2012) and expression of p75NGFR on GnRH cells low. These results indicate that p75NGFR is not involved in early, fast migration of GnRH neurons, but appears to inhibit migration as GnRH cells mature and approach their final destination.

A change in the morphology of GnRH neurons in the brain of p75NGF^−/−^ mice was found during prenatal development. GnRH cells were smaller than controls by ~30%. This difference was not found in GnRH cells in the adult brain nor in other neuropeptide cells in the hypothalamus of prenatal p75NGFR^−/−^ mice. Notably, prolonged perturbation with p75NGFR antibody blocker in nasal explants from 3 to 6div showed a change in GnRH cell morphology that was consistent with results in prenatal mutant mice. GnRH cells were smaller than controls by ~23%. However, chronic treatment with the antibody blocker also resulted in a loss of GnRH cells (~38%), specifically in the periphery of the explant. Loss of GnRH cells in p75NGFR^−/−^ mice was not found. Certainly, a loss of peptide production, delay in maturation or exit from the olfactory pit would also result in a decrease in GnRH cell number. However, the change observed in GnRH morphology detected during prenatal development in null mice is consistent with an “unhealthy”cell. Presently, it is unclear whether a compensatory mechanism is present *in vivo* that prevents GnRH “cell loss” and/or an indirect change occurred during chronic treatment *in vitro*.

p75NGFR binds all the known members of the neurotrophin family [nerve growth factor (NGF), brain-derived neurotrophin factor (BDNF), NT-3, and NT-4]. The fact that (1) ~38% of the GnRH population was absent in explants treated with anti-p75, (2) ~33% of GnRH cells in untreated explants expressed p75NGFR transcript, and (3) ~86% of these p75NGFR/GnRH cells also expressed TrKC, suggested a possible role for this signaling system in GnRH cells. Thus, chronic pharmacological manipulations were performed exposing explants to NT3 + p75NGFR blocker. In all aspects measured, GnRH cells in explants treated with NT3 + p75NGFR blocker showed changes similar to that found after treatment with p75NGFR blocker alone. Thus, addition of exogenous NT3 was unable to “rescue” the anti-p75NGFR effects on GnRH cells *in vitro*. A 50% loss of dorsal root ganglion sensory neurons occurs in p75 mutant mice (Bergmann et al., 1997) and lowering the levels of p75NGFR expression in sensory neurons *in vitro* with antisense oligonucleotides prevented survival when the neurons were taken from embryonic day 12 and 15 mice but not sensory neurons taken from E19 mice (Barrett and Bartlett, 1994). These data indicate that a switch in the role that p75NGFR plays in cell survival occurs over development. Characterization of p75NGFR expression in GnRH cells *in vitro* revealed p75NGFR expression increased as a function of time and distance the GnRH cells had migrated from the tissue mass. Thus, cells expressing highest levels of p75NGFR are those “lost” in chronic perturbation studies.

The p75NGFR^−/−^ mice showed reduction in GAD67 fibers at the NFJ. GAD67 fibers are associated with sensory cells in the developing vomeronasal organ and represent a subpopulation of axons within the dense olfactory bundles (Wray et al., 1996). A reduction in olfactory axons was not reported by Tisay et al. (2000) using Haematoxylin and eosin staining on prenatal sections and was not discernable in our studies when markers were used that highlighted numerous olfactory fibers such as peripherin and N-CAM (Wray et al., 1994). Attenuated fibers are consistent with earlier observations in p75NGFR^−/−^ mouse which showed DRG sensory neuron fibers stunted and poorly arborized compared to those of WT littermates (Bentley and Lee, 2000). Neurotrophins promote neurite growth by binding p75NGFR and Trk tyrosine kinases and activating several signal transduction pathways. In this context, p75NGFR not only mediates signals by itself to promote neurite growth but also increases the neurotrophin binding affinity to Trk and promotes Trk signaling (Lee et al., 2001). However, chronic treatment with p75NGFR antibody blocker showed a decrease in the density of peripherin-positive olfactory fibers with a corresponding increase in fiber complexity.

The p75NGFR^−/−^mice also showed reduction in GFAP-positive fibers at the NFJ. GFAP marks maturing OECs. OECs stimulate axon growth during development (Bentley and Lee, 2000) and promote axon regeneration when implanted into injured CNS (Raisman and Li, 2007). A defect in Schwann cell migration from the dorsal root ganglia has been reported in these mice (Bentley and Lee, 2000) and a link between Schwann cell migration and axon outgrowth was suggested. In a similar manner, a defect in OECs could alter olfactory sensory axon outgrowth and indirectly alter GnRH neuronal migration. A change in the morphology of OECs as a function of distance from tissue mass was detected in chronic *in vitro* perturbation studies. Compared to fusiform-shaped cells in the control group, OECs in the treated group became relatively flat with numerous processes. Whether this change relates to the decrease in GFAP staining detected in p75NGFR^−/−^ embryos is unclear. OECs secrete axon and neurite growth promoting extracellular matrix proteins such as laminin, fibronectin, type-IV collagen and cell adhesion molecules like NCAM and L1 (Doucette, 1990; Ramón-Cueto and Nieto-Sampedro, 1992). Thus, defasciculation of olfactory fibers documented *in vivo* in p75NGFR^−/−^ mice and in perturbation studies *in vitro* may be secondary to changes in OECs that express p75NGFRs.

Together, the data presented in this paper show that p75NGFR signaling is developmentally regulated in GnRH neurons and that signals through this receptor are important modulators of the olfactory/GnRH neuronal systems. Clearly, both direct and indirect actions of p75NGFR signaling could occur in this region as the development of the GnRH system and olfactory system are entwined.

### Conflict of interest statement

The authors declare that the research was conducted in the absence of any commercial or financial relationships that could be construed as a potential conflict of interest.
